# Challenges in the Estimation of the Annual Risk of *Mycobacterium tuberculosis* Infection in Children Aged Less Than 5 Years

**DOI:** 10.1093/aje/kwx153

**Published:** 2017-05-19

**Authors:** P Y Khan, Judith R Glynn, T Mzembe, D Mulawa, R Chiumya, Amelia C Crampin, Katharina Kranzer, Katherine L Fielding

**Keywords:** annual risk of tuberculous infection, bacille Calmette-Guérin, children, infection prevalence, Malawi, *Mycobacterium tuberculosis*, tuberculin skin test

## Abstract

Accurate estimates of *Mycobacterium tuberculosis* infection in young children provide a critical indicator of ongoing community transmission of *M. tuberculosis*. Cross-reactions due to infection with environmental mycobacteria and/or bacille Calmette-Guérin (BCG) vaccination compromise the estimates derived from population-level tuberculin skin-test surveys using traditional cutoff methods. Newer statistical approaches are prone to failure of model convergence, especially in settings where the prevalence of *M. tuberculosis* infection is low and environmental sensitization is high. We conducted a tuberculin skin-test survey in 5,119 preschool children in the general population and among household contacts of tuberculosis cases in 2012–2014 in a district in northern Malawi where sensitization to environmental mycobacteria is common and almost all children are BCG-vaccinated. We compared different proposed methods of estimating *M. tuberculosis* prevalence, including a method described by Rust and Thomas more than 40 years ago. With the different methods, estimated prevalence in the general population was 0.7%–11.5% at ages <2 years and 0.8%–3.3% at ages 2–4 years. The Rust and Thomas method was the only method to give a lower estimate in the younger age group (0.7% vs 0.8%), suggesting that it was the only method that adjusted appropriately for the marked effect of BCG-attributable induration in the very young.

Childhood tuberculosis has not been considered a priority in high-burden settings until recent years (1). Children have paucibacillary disease and are unlikely to contribute to onward transmission of *Mycobacterium tuberculosis* (2). This has led to significant underreporting of pediatric tuberculosis (1). However, childhood tuberculosis and *M. tuberculosis* infection in the very young necessarily result from recent transmission, so accurate estimates could provide a critical indicator of the effectiveness of prevention programs to curtail ongoing community *M. tuberculosis* transmission (3, 4).

Historically, measurements of the global burden of tuberculosis, including the incidence of tuberculosis disease, have been inferred in part from estimates of the annual risk of *M. tuberculosis* infection (ARTI) as derived from *M. tuberculosis* infection prevalence data obtained from tuberculin skin-test (TST) surveys in school-age children (5–7). Direct estimates of tuberculosis disease incidence would require prohibitively large longitudinal cohorts, even in areas where the burden of disease is high (8). Hence the comparatively inexpensive and logistically simple TST surveys were undertaken on a global programmatic scale. The inference of tuberculosis disease incidence from ARTI was based on the Styblo rule, where a 1% ARTI risk corresponds to 50 incident tuberculosis cases per 100,000 population per year (5). It is now recognized that accurate estimates of incidence of tuberculosis cases using the Styblo rule are not valid (9), although trend estimates of ARTI based on tuberculin surveys can be useful (10–13).

The TST measures the immunological response to a previously acquired infection with a mycobacterium that shares antigens with those in tuberculin. The challenge is to disentangle reactions due to *M. tuberculosis* infection from reactions due to exposure to environmental mycobacteria and bacille Calmette-Guérin (BCG) vaccination (12). Despite the lack of specificity of the TST (14), and because of the cost and logistical issues (need for venipuncture, skilled personnel, and laboratory equipment) (15) and the lack of clarity around the conversion and reversion phenomena associated with serial testing of the more specific interferon-gamma release assays (IGRAs) (16), serial population-wide tuberculin surveys undertaken in young children in high-burden countries remain among the few ways to assess the impact of tuberculosis-control strategies over time. However, this assessment relies on the need for a consistent estimate of the prevalence of *M. tuberculosis* infection, which is not always possible with the traditional cutoff methods, especially in settings where cross-reactivity with environmental mycobacteria and BCG-attributable reactions are common (11, 17).

Despite the advent of sophisticated statistical techniques, such as latent variable modeling (18), ascertainment of the prevalence of *M. tuberculosis* infection using tuberculin data is not always possible. Failures of the model to converge are frequent, especially in areas where there is a moderate to strong influence of cross-reactions and low prevalence of *M. tuberculosis* infection (12). An alternative method to estimate the prevalence of *M. tuberculosis* infection was published by Rust and Thomas 40 years ago (19), using tuberculin data from US Navy recruits. The authors stated that their proposed approach should “become even more preferable in the years to come” (p. 320-321, 19) because the prevalence of *M. tuberculosis* infection would continue to decrease compared with the prevalence of infection with environmental mycobacteria, which is likely to remain constant.

We aimed to determine the prevalence of *M. tuberculosis* infection and the ARTI in recently BCG-vaccinated preschool children in rural Malawi using the model proposed by Rust and Thomas. We compared these estimates with those derived using the classical TST cutoff methods (indurations of ≥10 mm or ≥15 mm), fixed-mirror method (6, 20), and mixture analysis (21–24).

## METHODS

### Study setting

Karonga district in northern Malawi is predominantly rural, with an adult human immunodeficiency virus (HIV) prevalence around 9% and incidence of new smear-positive tuberculosis of 87/100,000 adults per year (25). BCG vaccination is administered to children on first health system contact (usually birth) as part of the Expanded Program on Immunization. The whole population (approximately 39,000 people) in an area in the south of the district is under demographic surveillance in the Karonga Health and Demographic Surveillance System (KHDSS) (26).

### Study participants

#### Population at low risk of *M. tuberculosis* infection

We conducted a population-wide TST survey in preschool children in 2012, nested in the KHDSS. All children aged 3 months to 4 years, resident in the KHDSS area at the time of household recruitment, were eligible to take part in the study.

#### Population at high risk of *M. tuberculosis* infection

We also conducted a cross-sectional, household case-contact study of tuberculosis throughout the district from January 2013 to December 2014. Household contacts, including children aged <5 years, of an adult with smear-positive pulmonary tuberculosis were tuberculin tested.

### Study procedures

Field staff were trained in the placement and reading of skin tests according to standard international guidelines (27). Two international units of tuberculin purified protein derivative RT23 (Statens Serum Institut, Copenhagen, Denmark) were injected into the volar surface of the forearm, and induration was measured 48–72 hours later. The transverse and longitudinal diameters of the induration were recorded to the nearest millimeter, and an average was calculated (20).

Children with TST ≥10 mm were assessed for tuberculosis-related symptoms by field staff, and the results were recorded in the child's health passport. Any child with symptoms suggestive of tuberculosis (fever, weight loss, failure to thrive, night sweats, or cough) was reviewed by a clinician and referred to the district hospital. All children with TST ≥15 mm were commenced on 6-month isoniazid preventive treatment (10 mg/kg once daily) after active disease had been excluded.

Demographic data on sex, household size, household socioeconomic status (using a composite score based on quality of dwelling place, number of assets, employment of head of household, food security, and availability of soap), and maternal HIV status were collected for all study participants. HIV status of the child was not ascertained unless clinically indicated. Written informed consent was obtained from a parent or guardian of each participating child.

Both studies were approved by the Malawi National Health Sciences Research Committee (study protocols #944 and #1049) and the London School of Hygiene and Tropical Medicine ethics committee (study protocols #6065 and #6285).

### Statistical analyses

The frequency distributions of induration were tabulated using 2-mm categories to minimize the number of categories with no data. We used 2 categories: 1) population at higher risk of *M. tuberculosis* infection: children aged <5 years resident in the household of an adult with smear-positive pulmonary tuberculosis; and 2) population at lower risk of *M. tuberculosis* infection: children aged <5 years resident in the KHDSS, excluding 20 children who had known direct household contact with tuberculosis. We then used 4 methods to estimate the prevalence of *M. tuberculosis* infection in both populations:

#### Rust and Thomas method

This method is based on the distribution of induration size (mm) rather than a classification system of defining individuals as positive, negative, or doubtful. The technique was originally applied to a well-defined population of white male US Navy recruits aged 17–21 years, who had been lifetime US residents. This population was then divided into 2 groups, those with known household exposure to a person with tuberculosis, based on self-report (defined as “high risk”), and those without such exposure (defined as “low risk”).

The Rust and Thomas method is built on a simple mathematical model. The underlying assumptions are:

- The population can be divided into 2 groups that differ only in the prevalence of the infection.
- There is an identifiable category in which no individual has *M. tuberculosis* infection (TST = 0 mm).
- There is an identifiable category in which all individuals have *M. tuberculosis* infection (TST ≥ *n* mm).

The rationale of the Rust and Thomas model is as follows: In a hypothetical population without any *M. tuberculosis* infection, the majority will have a TST of 0-mm induration. If sensitization to environmental mycobacteria and/or recent BCG vaccination is prevalent, reactions of moderate size will also occur. The distribution of this population is called the “noninfected” distribution, referring to the absence of infection with *M. tuberculosis.* Comparably, in a hypothetical population in which everyone has been infected with *M. tuberculosis*, all but a few individuals will have a fairly large reaction size, and a bell-shaped “infected” distribution will be observed. In an existent population, the observed distribution will be a combination of “infected” and “noninfected” distributions. Thus, the observed “higher-risk” and “lower-risk” groups are each a mixture of overlapping distributions of “infected” and “noninfected”. If one observed the “noninfected” population alone, there would be a very large proportion with zero induration, and the proportion of “noninfected” with small to medium-sized reactions would depend on the prevalence of nontuberculous mycobacterial infection and/or BCG-attributable induration (i.e., the distribution of reaction size depends upon sensitization to environmental mycobacteria or recent BCG vaccination but not upon contact status). Expressed in a different way, the ratio of the proportion with zero induration to the proportion “noninfected” is constant (see equation 1 below) (19).

Similarly, the distribution of reaction sizes among those who are truly infected is independent of contact status. Assuming that there is a reaction size (*n*) above which all individuals are infected, the ratio of the proportion in category *n* to the proportion “infected” is constant (see equation 2 below).

Equation 1 for a “noninfected” population:

(1) $$\frac{f_{0}}{1-P}=\frac{{f\prime }_{0}}{1-P\prime }$$

and equation 2 for an “infected” population:

(2) $$\frac{f_{n}}{P}=\frac{{f\prime }_{n}}{P\prime },$$

where *f*_0_ = proportion of higher-risk population with zero induration, ${f\prime }_{0}$ = proportion of lower-risk population with zero induration, *f*_n_ = proportion of higher-risk population in induration category *n*, f′ = proportion of lower-risk population in induration category *n*, *P* = prevalence of *M. tuberculosis* infection in the higher-risk population, and *P*′ = prevalence of *M. tuberculosis* infection in the lower-risk population.

Equations 1 and 2 can then be solved for *P* and *P*′, the prevalence of *M. tuberculosis* infection in the higher-risk and lower-risk groups, respectively.

$$P=\frac{f_{n}({f\prime }_{0}-f_{0})}{f_{n}{f\prime }_{0}-{f\prime }_{n}f_{0}}$$

$$P\prime =\frac{{f\prime }_{n}({f\prime }_{0}-f_{0})}{f_{n}{f\prime }_{0}-{f\prime }_{n}f_{0}}$$

A TST reaction size of ≥20 mm was chosen as the category *n* in which all individuals were assumed to have *M. tuberculosis* infection. This category was chosen following examination of the prevalence of infection calculated for different values of *n*. The optimal choice was that category *n* in which the computed prevalence is approximately the same as that for higher values of *n* (19). (See Web Table 1 and Web Appendix 1, available at https://academic.oup.com/aje, for details on the selection of the reaction size of 20 mm).

Bias-corrected 95% confidence intervals were calculated using a bootstrapping approach in Stata, version 14.1 (StataCorp LP, College Station, Texas).

#### Fixed cutoff points at 10 mm or 15 mm

*M. tuberculosis* infection prevalence was calculated as the proportion of children with a “positive” TST defined by cutoff points at ≥10 mm or ≥15 mm, divided by the total number of children with a TST result.

#### Fixed-mirror method (17 mm)

The fixed-mirror method assumes that among individuals with *M. tuberculosis* infection, the distribution of induration size is symmetric around a fixed mode of 17 mm, and that no nonspecific reactions, such as BCG-attributable induration, reach 17 mm (6, 20). Therefore all reactions of 17 mm were counted once, and indurations of >17 mm were counted twice and summed to obtain the estimated number of *M. tuberculosis* infections. Prevalence was calculated as the count of “*M. tuberculosis* infections” divided by the total number of children with TST results (24).

#### Mixture analysis

Mixture analysis of the tuberculin survey data, which is a form of latent variable modeling (18), was based on implementation of the Bayesian Markov Chain Monte Carlo approach in R (R Foundation for Statistical Computing, Vienna, Austria) (28). Three parametric models (Weibull, log-normal, and gamma distributions) were tested to determine the best-fitting model using the maximum log likelihood function as a guide. The quality of the fit was assessed by comparing predicted and observed frequencies via posterior predictive model checks (24, 28).

### Sensitivity analyses

The effect of neonatal BCG vaccination on TST induration, which is most pronounced in the first few months after vaccination, is thought to wane rapidly (29, 30). The analysis was repeated, stratifying children into age groups of <2 years and 2–4 years, to assess the effect of BCG-attributable induration on estimates of *M. tuberculosis* prevalence.

### Annual risk of *M. tuberculosis* infection

The ARTI, the probability of being infected in any one year, was calculated using the formula (31):

$$\mathrm{ARTI}\approx 1-{\left(1-P\right)}^{1/a}$$

where *P* is the prevalence of *M. tuberculosis* infection, and *a* is the mean age of children. The ARTI was calculated only for the children resident within the KHDSS, which was assumed to be representative of the ARTI in children aged <5 years in the district.

## RESULTS

The frequency distribution of tuberculin data from the lower-risk and higher-risk study populations are shown in Table 1 and the Web Figures 1–4. Among all children <5 years of age, 85% of the lower-risk population had zero induration compared with 56% of the higher-risk population (*P* < 0.001). When stratifying by age, the proportion with zero induration in the lower-risk group was 92% in those aged 2–4 years compared with 73% in those aged <2 years. In the higher-risk group the proportion with zero induration was 54% in those aged 2–4 years and 60% in those <2 years. There was no evidence that distribution of induration size was affected by the HIV-exposure status of the child (χ^2^ test: lower-risk group, *P* = 0.9; higher-risk group, *P* = 0.8).

**Table 1.** Frequency Distribution of Tuberculin Data in Rural Children Aged Less Than 5 Years Stratified by Risk Group and Age, Karonga District, Malawi, 2012–2014

Induration Size, mm	Frequency Distribution of Tuberculin Data											
	Lower-Risk Group						Higher-Risk Group					
	All Aged <5 Years (*n* = 4,947)		Aged <2 Years (*n* = 1,797)		Aged 2–4 Years (*n* = 3,150)		All Aged <5 Years (*n* = 152)		Aged <2 Years (*n* = 52)		Aged 2–4 Years (*n* = 100)	
	No.	%	No.	%	No.	%	No.	%	No.	%	No.	%
0	4,187	84.7	1,301	72.4	2,886	91.6	85	55.9	31	59.6	54	54.0
2–3	26	0.5	10	0.6	16	0.5	3	2.0	1	1.9	2	2.0
4–5	72	1.4	37	2.1	35	1.1	2	1.3	2	3.8	0	0.0
6–7	160	3.2	105	5.8	55	1.7	4	2.6	3	5.8	1	1.0
8–9	191	3.9	137	7.6	54	1.7	2	1.3	1	1.9	1	1.0
10–11	99	2.0	60	3.3	39	1.2	4	2.6	1	1.9	3	3.0
12–13	88	1.8	62	3.5	26	0.8	8	5.3	3	5.8	5	5.0
14–15	64	1.3	49	2.7	15	0.5	9	5.9	2	3.8	7	7.0
16–17	32	0.6	22	1.2	10	0.3	11	7.2	2	3.8	9	9.0
18–19	19	0.4	9	0.5	10	0.3	13	8.6	2	3.8	11	11.0
20–21	3	0.1	1	0.1	2	0.1	8	5.3	3	5.8	5	5.0
≥22	6	0.1	4	0.2	2	0.1	3	2.0	1	1.9	2	2.0

### Prevalence of *M. tuberculosis* infection

Table 2 shows the estimated prevalence of *M. tuberculosis* infection using the different methods. In the lower-risk group the estimates of infection prevalence were consistently higher among children less than 2 years of age compared with those aged 2–4 years using all methods except for the Rust and Thomas model. For children less than 2 years of age, the estimates ranged from 0.7% to 11.5%; the mixture model and the TST (≥10-mm cutoff) method estimated the highest infection prevalence (11%–12%). Although the fixed-mirror method and the Rust and Thomas method estimated similar infection prevalences for children 2–4 years of age, among children less than 2 years of age the infection prevalence estimate using the fixed-mirror method was nearly 3 times that of the Rust and Thomas method.

**Table 2.** Prevalence Estimates of *Mycobacterium tuberculosis* Infection Using Different Methods in Rural Children Aged Less Than 5 Years, Karonga District, Malawi, 2012–2014

Estimation Method	Lower-Risk Group						Higher-Risk Group					
	All Aged <5 Years (*n* = 4,947)		Aged <2 Years (*n* = 1,797)		Aged 2–4 Years (*n* = 3,150)		All Aged <5 Years (*n* = 152)		Aged <2 Years (*n* = 52)		Aged 2–4 Years (*n* = 100)	
	%	95% CI	%	95% CI	%	95% CI	%	95% CI	%	95% CI	%	95% CI
TST cutoff, mm												
≥10	6.3	5.6, 7.0	11.5	10.1, 13.1	3.3	2.7, 4.0	38.6	30.3, 47.5	26.9	15.6, 41.0	41.8	31.5, 52.6
≥15	1.9	1.6, 2.4	3.4	2.6, 4.3	1.1	0.8, 1.5	28.8	21.2, 37.3	17.3	8.2, 30.3	31.9	22.5, 42.5
Fixed-mirror method	1.3	1.0, 1.7	2.0	1.4, 2.7	1.0	0.7, 1.4	36.8	29.2, 45.0	26.9	15.6, 41.0	42.0	32.2, 53.3
Mixture model	6.4	3.4, 9.9	12.4	4.4, 19.3	1.9	0.03, 4.7	33.4	24.2, 43.1	27.6	4.4, 47.8	39.9	24.4, 49.9
Rust and Thomas model	0.9	0.3, 2.4	0.7	0.1, 4.6	0.8	0.2, 2.6	34.5	25.6, 43.8	18.2	0.0, 35.8	41.5	30.2, 51.8

Abbreviations: CI, confidence interval; TST, tuberculin skin test.

In the higher-risk group the estimates were higher among children aged 2–4 years than among the youngest age group for all methods. The estimates for the older age group in the higher-risk group were similar for all methods, ranging from 39.9% to 42.5%, except for the TST (≥15-mm cutoff) method, which estimated a prevalence of *M. tuberculosis* infection of 32%. The bias-corrected 95% confidence interval of Rust and Thomas estimates for the higher-risk children less than 2 years of age includes 0. This is likely to be a result of the small sample size, *n* = 52, in this group.

### Annual risk of *M. tuberculosis* infection

ARTI estimates ranged from 0.3% (95% CI: 0.1, 0.9) to 2.6% (95% CI: 1.8, 2.7) depending on the method used to estimate the prevalence of *M. tuberculosis* infection. The Rust and Thomas model estimate was the most conservative at 0.3% (95% CI: 0.1, 0.9). It was also the method that demonstrated the least difference in ARTI estimates when stratified by age (see Table 3).

**Table 3.** Estimates of Annual Risk of *Mycobacterium tuberculosis* Infection Based on Prevalence Estimates of *M. tuberculosis* Infection in the Children Under 5 Years of Age Resident in a Demographic Surveillance Area, Karonga District, Malawi, 2012–2014

Method	Annual Risk of *M. tuberculosis* Infection					
	All Aged <5 Years		Aged <2 Years		Aged 2–4 Years	
	%	95% CI	%	95% CI	%	95% CI
TST cutoff, mm						
≥10	2.4	1.8, 2.7	10.2	6.3, 11.6	1.0	0.7, 1.2
≥15	0.7	0.5, 0.8	3.0	2.3, 3.8	0.3	0.2, 0.4
Fixed-mirror method	0.5	0.4, 0.7	1.8	1.2, 2.4	0.3	0.2, 0.4
Mixture model	2.6	1.4, 4.0	10.5	3.2, 17.1	0.6	0.1, 1.4
Rust and Thomas model	0.3	0.1, 0.9	0.6	0.1, 4.1	0.2	0.1, 0.8

Abbreviations: CI, confidence interval; TST, tuberculin skin test.

## DISCUSSION

Our findings highlight the challenges of using tuberculin surveys to estimate the risk of *M. tuberculosis* infection in young BCG-vaccinated children. ARTI estimates varied 5-fold depending on the method used to estimate *M. tuberculosis* infection prevalence. The Rust and Thomas method generated a consistent estimate of infection prevalence and ARTI, irrespective of age, in a setting where sensitization to environmental mycobacteria is known to be high (22) and over 90% of children are BCG-vaccinated within 3 months of birth. It was the only method that appeared to adjust appropriately for the marked effect of BCG-attributable induration in the very young (aged <2 years).

The Rust and Thomas method protects against changes in prevalence estimates caused by differences in strength of tuberculin used or the use of different equipment and/or techniques, thus making it possible to compare *M. tuberculosis* infection prevalence found by different investigators at varying times and places (19). Because the Rust and Thomas method relies on the distribution of induration in those known to have been exposed to *M. tuberculosis* and the distribution of induration in those at “lower” risk at the same point in time, as long as the same tuberculin and technique is used in both populations, the prevalence estimates over time are much more likely to be comparable, despite differences in geographical settings, climate zones, changing BCG vaccination policies, and introduction of new vaccines. In addition, the Rust and Thomas model can be used to generate the probability of *M. tuberculosis* infection at each induration size, thereby making it possible to calculate sensitivity and specificity, area under receiver operating characteristic curve, and the positive predictive value of the TST in a given population (14). Another advantage compared with traditional cutoff methods is that prevalence estimates are less sensitive to alterations in the chosen critical point. For the Rust and Thomas method, this is the reaction-size category in which all individuals are assumed to have *M. tuberculosis* infection. As long as this reaction size exceeds the maximum reaction size occurring among the “noninfected,” the calculated prevalence will be subject only to random fluctuations. However, if the reaction size is too small, the basic assumption that all individuals with reactions of that size or larger have been infected with *M. tuberculosis* will not be fulfilled, and the estimated infection prevalence will therefore overestimate the true prevalence (19).

One of the reasons the Rust and Thomas method has been apparently forgotten may be the requirement of tuberculin data from “low-risk” and “high-risk” groups. The assessment of US Navy recruits, used in the original study, used self-report of household contact with an individual with tuberculosis (32). In our study, we combined tuberculin data from a TST survey conducted in a demographic surveillance area with data from a concurrent case-contact household study of tuberculosis in the whole district. The demographic surveillance area may not be representative of the whole district: Research has been conducted in KHDSS for the last 12 years, which may have influenced health-seeking behavior, which in turn may affect *M. tuberculosis* transmission dynamics in the area. One of the major assumptions of the Rust and Thomas model is that the “high risk” and “low risk” populations differ only with respect to contact status and therefore prevalence of infection. Reassuringly, the 2 groups in our study did not differ significantly with regard to age, sex, household size, household socioeconomic status, and maternal HIV status (see Web Appendix 2 and Web Table 2).

In the lower-risk group in our study, the proportion of children with a TST ≥20 mm (our chosen *n*th category) was larger among children under 2 years of age (0.5%) than among those of ages 2–4 years (0.2%). If this is not due to *M. tuberculosis* infection, it would violate the assumption that only those truly infected are included in the *n*th category and would therefore overestimate the infection prevalence. Similarly, for the fixed-mirror method, any induration size ≥17 mm due to BCG-attributable induration rather than true *M. tuberculosis* infection prevalence would overestimate infection prevalence. Very large induration secondary to BCG vaccination is more likely to occur among children less than 2 years of age who have been more recently vaccinated. Interestingly, a Taiwanese study that proposed age-specific cutoffs to detect *M. tuberculosis* infection in children suggested a cutoff of 21 mm for infants aged less than 2 years (33).

Data in the larger-induration (>20 mm) categories were sparse, and misclassification of a small number has a large effect on the resultant proportion in the *n*th category, which is a limitation of the data. The 95% confidence interval of the prevalence of *M. tuberculosis* infection among the higher-risk children aged <2 years using the Rust and Thomas method included 0, also underscoring the importance of an adequate sample size. A similar study among older children, adolescents, and young adults—who are likely to be at greater risk for *M. tuberculosis* infection than are young children (20, 34, 35)—would be useful to assess the robustness of the Rust and Thomas method. It would require a household contact study as well as a “low-risk” population survey, which would have cost implications.

Our findings present evidence that the Rust and Thomas method appears to address the effect of recent BCG vaccination among children under 2 years of age. Among the older children, the results of the different methods vary less and are all likely to be plausible, but because the Rust and Thomas method performed more appropriately in dealing with the cross-reactions due to BCG in the younger age group, we can have confidence that it is dealing appropriately with cross-reactivity in the older age group as well.

One might ask why we should continue to advocate the use of tuberculin in an era of more specific diagnostics, such as IGRAs, and newer skin tests, such as the C-Tb skin test, a novel skin test containing ESAT-6 and CFP-10, antigens that are specific to *M. tuberculosis* (36). The latest skin tests, for which there are currently limited data, do appear to offer higher specificity than tuberculin, but this might come at the cost of reduced sensitivity (37). The cost, technical complexity, and the requirement of laboratory infrastructure in order to undertake large IGRA surveys usually preclude population-level studies. However, IGRA substudies nested within tuberculin surveys could potentially be used to refine estimates of *M. tuberculosis* infection prevalence (12, 38, 39), although it is not clear how discrepancies between TST and IGRA should be interpreted. In longitudinal studies, IGRA and TST responses seem to convert and revert at different rates, so the 2 tests are unlikely to give the same assessment of infection in any population (40, 41).

We wanted to estimate the ARTI in preschool children based on the rationale that determination of the average ARTI in the very young provides a critical indicator of the extent of recent *M. tuberculosis* transmission. It is important to bear in mind that risk of *M. tuberculosis* infection is not independent of age (20, 34, 42) and is most likely related to *M. tuberculosis* exposure through age-assortative social mixing (43). Thus the average ARTI in the youngest within a population is unlikely to be representative of the ARTI in those that are older, but it does provide the most contemporary marker of recent *M. tuberculosis* transmission. Repeated tuberculin surveys in the youngest generation could potentially be used to assess whether implementation of tuberculosis-control strategies within the community have resulted in a decrease of recent *M. tuberculosis* transmission (11).

In conclusion, there is unequivocally a need for more accurate epidemiologic indicators of *M. tuberculosis* transmission and *M. tuberculosis* infection prevalence estimates in order to understand the dynamics of tuberculosis epidemiology in varying settings (44). In our study, the Rust and Thomas method was the only method to find a lower estimate in the youngest age group, suggesting that it accounted appropriately for the cross-reactivity due to BCG vaccination.

## Supplementary Material

Web Material
