# Supplementary material for: Challenges in the Estimation of the Annual Risk of Mycobacterium tuberculosis Infection in Children Aged Less Than 5 Years
Source: Am J Epidemiol. 2017 May 19;186(8):1015–22. doi: 10.1093/aje/kwx153 (PMC5784799; doi:10.1093/aje/kwx153)
Supplement: Web Material [file kwx153khanwebmaterialfinal.pdf]

Web Material

Web Table 1. *M.tb* Infection Prevalence (%) (P and P′) Using Different Induration Categories for *n*

| | | Induration (mm) considered to identify *M.tb* infection: | | | | | | |
|---|---|---|---|---|---|---|---|---|
| | | **22+** | **20+** | **18+** | **16+** | **14+** | **12+** | **10+** |
| | All < 5 | 35.9 | 34.5 | 34.8 | 35.2 | 36.0 | 37.0 | 38.3 |
| ‘Higher risk’ group (years) | < 2 | 19.5 | 18.2 | 18.7 | 19.8 | 22.1 | 24.1 | 27.2 |
| | ≥ 2 | 43.4 | 41.5 | 41.7 | 41.8 | 42.0 | 42.4 | 43.1 |
| ‘Lower risk’ group (years) | All < 5 | 2.9 | 0.9 | 1.2 | 1.8 | 3.1 | 4.6 | 6.5 |
| | < 2 | 2.3 | 0.7 | 1.3 | 2.6 | 5.5 | 7.9 | 11.7 |
| | ≥2 | 3.9 | 0.8 | 1.0 | 1.2 | 1.6 | 2.3 | 3.4 |
| | 2.0 - 2.9 | 3.9 | 1.6 | 0.6 | 0.6 | 0.8 | 1.3 | 2.6 |
| | 3.0 – 3.9 | - | - | 1.2 | 1.2 | 1.4 | 1.8 | 3.0 |
| | 4.0 – 4.9 | - | 1.2 | 1.2 | 1.8 | 2.6 | 3.9 | 4.4 |

Web appendix 1

The prevalence of *M.tb* infection for each group was calculated for different reaction size categories using the equations for P and P′ (see Methods) and are shown in table above (Web table 1). Infection prevalence estimates start to become ‘approximately constant’, as deduced from visual inspection, from 16+ category in all groups apart from the under-2s in the ‘lower risk’ group. This suggests that the 16+ category in the ‘lower risk’ under-2s includes individuals who are not truly ‘infected’. In order to fulfil the assumption that all individuals in category *n* are ‘infected’, one must choose the largest induration size allowed by the data. In this dataset, reaction size of 20mm was chosen to represent *n* category as there was obvious instability of estimates for the 22+ category seen across all groups due to the small numbers in this category.

Web Figure 1. Histogram illustrating the distribution of non-zero TST data in all children aged < 5 years stratified by risk group ('lower risk' N=4967; 'higher risk' N=152)

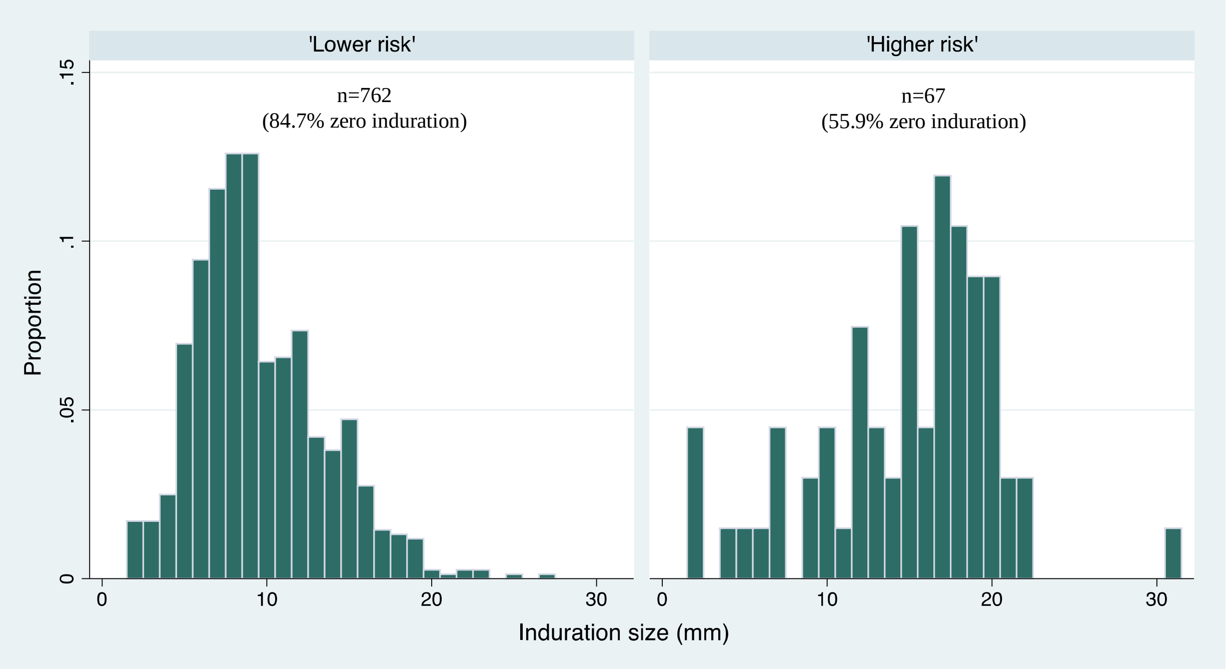


Web Figure 2. Histogram illustrating the distribution of non-zero TST data in children in the 'lower risk' group stratified by age (<2 years N=1797; ≥2 years N=3170)

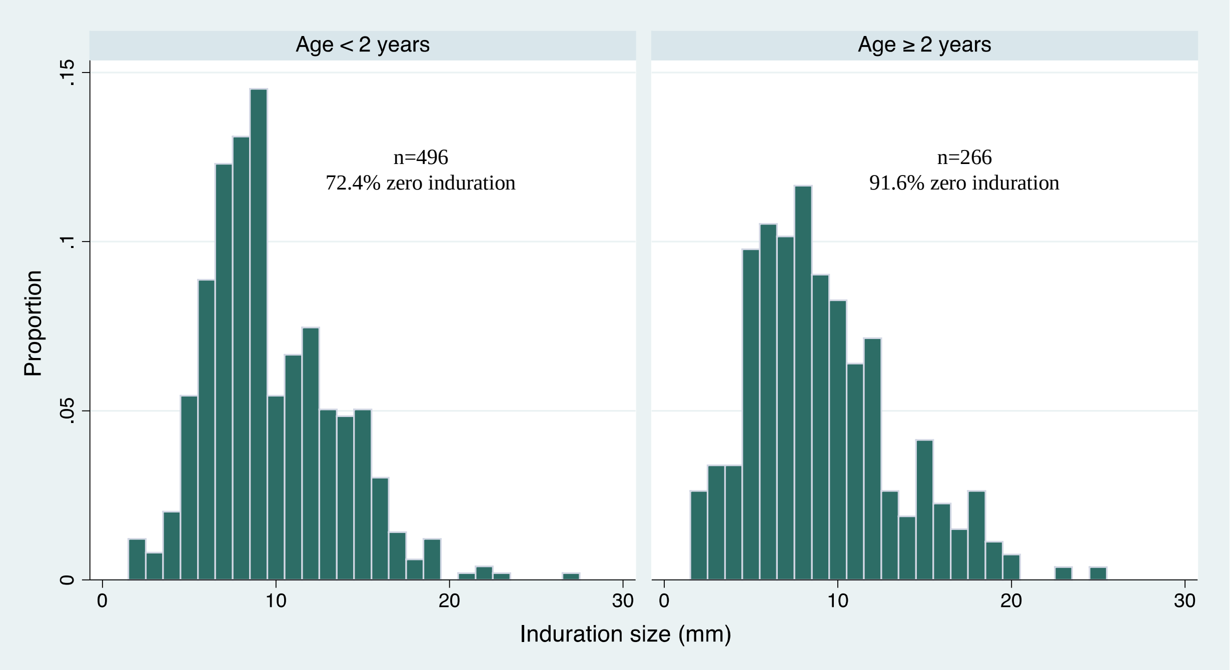

Web Figure 3. Histogram illustrating the distribution of non-zero TST data in children in the 'higher risk' group stratified by age (<2 years N=52; ≥2 years N=100)

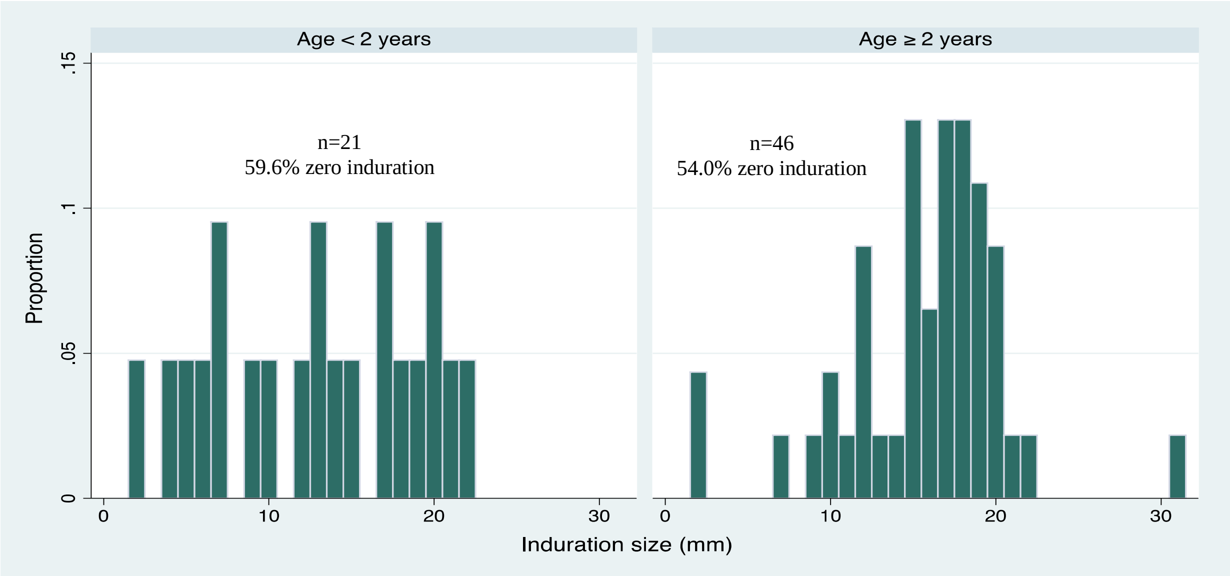


Web figure 4. Percentage distribution of induration category stratified by HIV exposure status and contact status of children ('Lower' risk not HIV-exposed n=4453; HIV-exposed n=272; 'higher' risk not HIV-exposed n=98; HIV-exposed n=17)

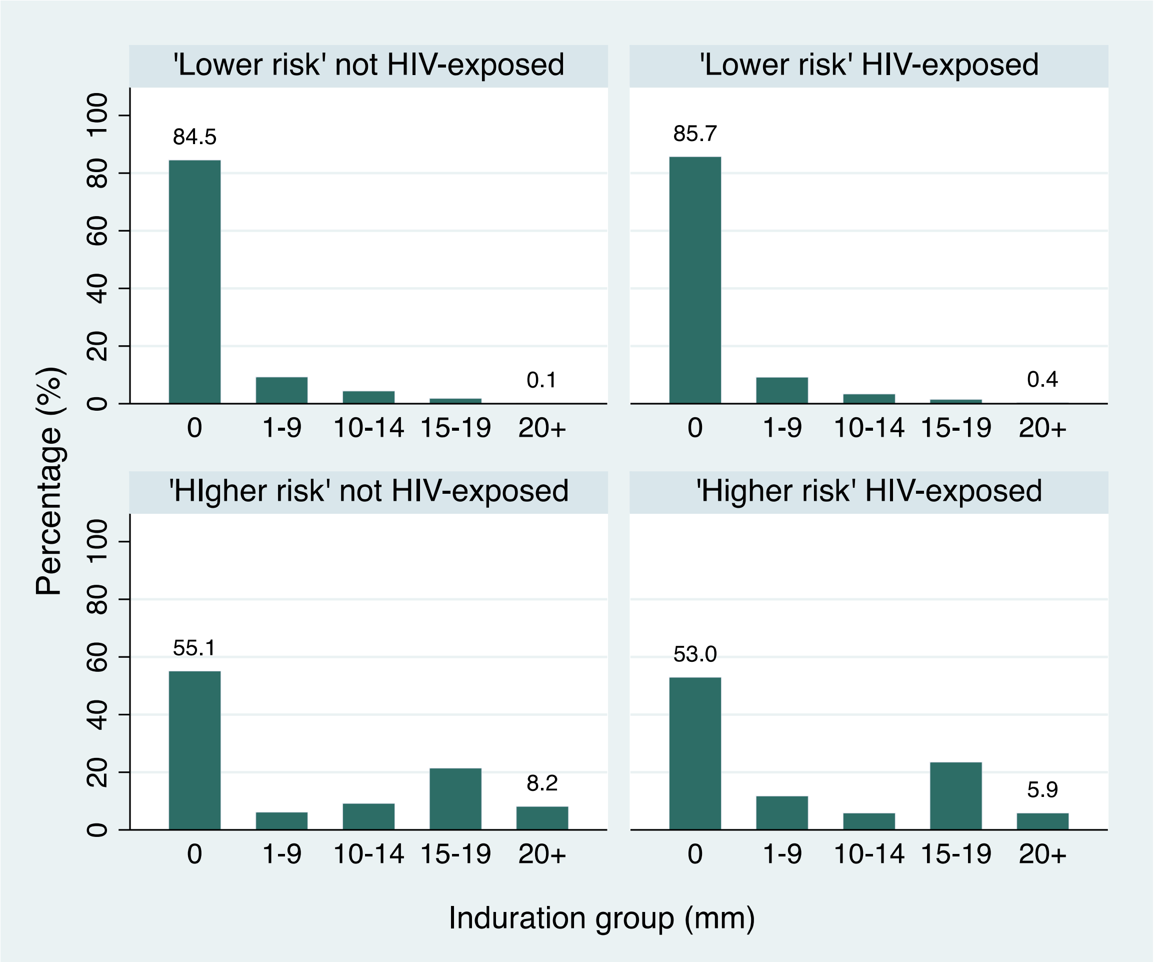

Web appendix 2

The Rust and Thomas method depends on the assumption that the two groups only differ with respect to contact status and prevalence of *M.tb* infection. Table (ii) shows the demographic characteristics of the 'low risk' and 'high risk' group.

Web table 2. Demographic Characteristics of the 'Lower Risk' and 'Higher Risk' Groups

| | 'Lower risk' | 'Higher risk' |
|---|---|---|
| Age in years (mean (sd)) | 2.6 (1.4) | 2.7 (1.3) |
| Male (%) | 50.0 | 48.5 |
| Mother HIV positive (%) | 5.5% (95% CI 4.9 – 6.2) | 11.2% (95% CI 6.6 – 17.3) |
| Median no. of adults in the household (IQR) | 2 (2 - 3) | 2 (1 - 2) |
| Proportion in the lowest SES category | 28.4% | 23.4% |

sd standard deviation; IQR interquartile range; CI confidence interval SES socioeconomic status
